# Eosinophils and Purinergic Signaling in Health and Disease

**DOI:** 10.3389/fimmu.2020.01339

**Published:** 2020-07-08

**Authors:** Davide Ferrari, Marta Vuerich, Fabio Casciano, Maria Serena Longhi, Elisabetta Melloni, Paola Secchiero, Andreas Zech, Simon C. Robson, Tobias Müller, Marco Idzko

**Affiliations:** ^1^Section of Microbiology and Applied Pathology, Department of Life Science and Biotechnology, University of Ferrara, Ferrara, Italy; ^2^Department of Anesthesia, Critical Care & Pain Medicine, Beth Israel Deaconess Medical Center, Harvard Medical School, Boston, MA, United States; ^3^Department of Morphology, Surgery and Experimental Medicine and LTTA Centre, University of Ferrara, Ferrara, Italy; ^4^Department of Pulmonology, Medical University of Vienna, Vienna, Austria; ^5^Division of Pneumology, University Hospital RWTH Aachen, Aachen, Germany

**Keywords:** eosinophils, extracellular ATP, extracellular adenosine, CD39, CD73, P1 receptors, P2 receptors, inflammation

## Abstract

Eosinophils are major effector cells against parasites, fungi, bacteria, and viruses. However, these cells also take part in local and systemic inflammation, which are central to eczema, atopy, rhinitis, asthma, and autoimmune diseases. A role for eosinophils has been also shown in vascular thrombotic disorders and in cancer. Many, if not all, above-mentioned conditions involve the release of intracellular nucleotides (ATP, ADP, UTP, etc.) and nucleosides (adenosine) in the extracellular environment. Simultaneously, eosinophils further release ATP, which in autocrine and paracrine manners, stimulates P2 receptors. Purinergic signaling in eosinophils mediates a variety of responses including CD11b induction, ROI production, release of granule contents and enzymes, as well as cytokines. Exposure to extracellular ATP also modulates the expression of endothelial adhesion molecules, thereby favoring eosinophil extravasation and accumulation. In addition, eosinophils express the immunosuppressive adenosine P1 receptors, which regulate degranulation and migration. However, pro-inflammatory responses induced by extracellular ATP predominate. Due to their important role in innate immunity and tissue damage, pharmacological targeting of nucleotide- and nucleoside-mediated signaling in eosinophils could represent a novel approach to alleviate eosinophilic acute and chronic inflammatory diseases. These innovative approaches might also have salutary effects, particularly in host defense against parasites and in cancer.

## Introduction

Nucleotides and nucleosides are present at high concentrations within the cell where they exert multiple functions. However, they are not restricted to the intracellular compartments but they serve as extracellular mediators to eukaryotic cells (1, 2). A growing body of evidence indicates that released nucleotides represent important modulators to several cell and organ pathways under both physiological and pathological conditions. Their role in the cardiocirculatory and the nervous system, in tissue metabolism, respiration and immune function, as well as in gastrointestinal and hepatic disease pathogenesis has been described recently (3–7).

Extracellular purines and pyrimidines have been implicated in the regulation of ciliary beat frequency, chloride/liquid secretion, goblet cell degranulation, epithelial mucus secretion, transmission of the respiratory nervous stimuli and modulation of the airway vascular tone (8–10). Accordingly, inhaled ATP is a powerful bronchoconstrictor in both healthy and asthmatic individuals. Furthermore, extracellular ATP can act as a damage-associated molecular pattern molecule (DAMP, also known as alarmin or danger molecule) to activate the inflammasome with subsequent upregulation of IL-1β, IL-18, and release of other pro-mobilizing mediators like high molecular group box 1 (Hmgb1) and S100 calcium-binding protein A9 (S100A9) (11).

Under homeostatic conditions extracellular ATP levels are rather low. This is due to a moderate release and rapid degradation by extracellular ATP-metabolizing enzymes (ectonucleotidases) (12, 13). However, in the course of infection, inflammation, hypoxic conditions due to ischemia as well as necrotic and apoptotic cell death ATP is released from intracellular storage pools and can reach a concentration high enough to be sensed by surrounding cells expressing P2 receptors (14–16). Besides the unregulated ATP release as a consequence of cell damage, mediated secretion of this extracellular messenger occurs through plasma membrane molecules such as connexins, pannexins, and P2X7 receptors (17–20). Apart from ATP, uridine nucleotides (UTP, UDP and UDP-glucose) can also be released in the extracellular space (21).

Eosinophils are polymorphonuclear cells mainly involved in the immune defense, tissue remodeling and inflammation. Activation and migration of these cells to inflammatory sites are crucial to tissue defense. In addition to the classical immune activators (chemokines, cytokines, microbial products, allergens, complement components) eosinophils are also capable to sense nucleotides that can amplify responses induced by other stimuli (22). Thus, extracellular nucleotides contribute to eosinophilic inflammation and tissue damage both in human and animal models (23, 24). Therefore, nucleotides and nucleosides are under intense investigation for their capacity to activate and recruit eosinophils. In this regard, high levels of ATP are present in the bronchoalveolar lavage fluid of patients suffering from eosinophilic pneumonia. This mediator also correlates with uric acid and IL-33 concentration (24, 25).

## P2 Receptors

P2 receptors are plasma membrane receptors for extracellular nucleotides. On the basis of cloning, functional and pharmacological data, two P2 receptor subfamilies have been described: P2X and P2Y receptors (2, 26). Differences in nucleotide sensitivity and specificity of the P2 receptor subtypes, allow the activation of distinct P2 receptor subsets depending on the nucleotide concentration and kind.

The P2X receptor subfamily represents ligand-gated ion channels selective for monovalent and divalent cations. These ion channels are homo- or in some cases hetero-multimers with carboxyl- and amino-terminal cytoplasmatic domains (27, 28). In mammals, seven different subunits have been identified and named P2X1-P2X7. Extracellular ATP is an agonist for all P2X subtypes and regulates their permeability to Na^+^, K^+^, Ca^2+^, Mg^2+^. While the majority of P2X receptors is rapidly desensitized (e.g., P2X1 and P2X3), the non-desensitizing P2X7 represents a peculiar subtype having a long carboxyl-terminal domain allowing the receptor to undergo a permeability transition from a plasma membrane channel to a large plasma membrane pore depending on ATP concentration and the way of stimulation (26).

Stimulation of the P2X7 subtype by high ATP concentrations is associated with a permeability transition due to the opening of a membrane pore with a cut-off of 900 Da (27). Transmembrane ion fluxes, driven by pore opening, induce transcription and secretion of different inflammatory cytokines such as IL-1β, IL-18, IL-6 (23). Pharmacological blocking, genetic ablation and attenuation of P2X7 function resulted in reduced inflammatory responses (29–31).

The P2Y receptors are seven transmembrane G-protein-coupled receptors with an extracellular amino-terminus and an intracellular carboxyl-terminus. Eight human P2Y subtypes have been identified and named: P2Y1, P2Y2, P2Y4, P2Y6, P2Y11, P2Y12, P2Y13 and P2Y14 (32). They differ in agonist specificity, coupled G-protein and transduced intracellular signaling. However, according to amino acid homology and presence of conserved motifs in the transmembrane α-helix 7, two groups have been described. The first group includes the P2Y1, P2Y2, P2Y4, P2Y6, and P2Y11 subtypes, having 25–52% amino acid identity and a Y-Q/K-X-X-R motif in the transmembrane α-helix 7 (33).

To the second group belong P2Y12, P2Y13 and P2Y14, with sequence homology of 47–48% and the presence of the K-E-X-X-L motif (2). Some evidence suggests that the two P2Y subgroups differ in G-protein coupling. Hence, the receptors of the first group couple to Gq/G11proteins, contributing to calcium release via phospholipase C/inositol-1,4,5-triphosphate activation; while receptors of the second group couple to Gi/0 proteins, inhibiting adenylate cyclase (AC) (34). Different P2Y agonists have been identified, among them both adenine and uridine nucleotides (35). P2Y1, P2Y12, and P2Y13 subtypes are preferentially activated by ADP (36), whereas UDP is an agonist at P2Y6. While P2Y2 can be activated by both UTP and ATP, P2Y4 and P2Y11 are selective for UTP and ATP, respectively. Last but not least, P2Y14 is activated by UDP-glucose (35).

In the last two decades, P2 receptors gained attention for their wide tissue distribution and number of modulated pathophysiological responses. This has also prompted several P2-based therapeutic approaches (37), as in the context of kidney disease, cardiovascular and metabolic disorders as well as central nervous system (CNS) inflammation.

## P1 Receptors

Similar to the P2Y receptors, the P1 receptors are seven-transmembrane G-protein-coupled receptors but their natural agonist is adenosine (ADO) (38). ADO exerts ambiguous effects in different tissues, depending on cell type and P1 receptor subtypes predominantly expressed (39–41).

Four receptors subtypes have been identified and named: A1 (ADORA1), A2A (ADORA2A), A2B (ADORA2B), and A3 (ADORA3), respectively. The main differences between the subtypes concern the affinity to ADO, the coupled G-protein families and effects on AC. While A1 and A3 inhibit AC, A2A and A2B drive its activation (38). ADO concentration of the extracellular *milieu* ranges from 100 to 500 nM and increases to levels in the low micromolar range as a consequence of inflammation, hypoxia and ischemia. Among the subtypes, A2B shows the lowest affinity for ADO. Accordingly, A1, A2A and A3 are activated by lower ADO concentrations (10–50 nM); whereas A2B needs a rather high agonist concentration (1 mM) for stimulation.

Primarily, adenosine receptors have been associated with dampening acute inflammation and tissue injury. On the one hand the inhibition of pro-inflammatory cytokine production and on the other hand the induction of suppressive cytokines as well as regulatory immune cell differentiation are two known effects of the anti-inflammatory responses driven by P1 receptors. Nevertheless, in the context of rheumatoid arthritis or multiple sclerosis P1 receptors have also been implicated in inflammatory cell recruitment (42–44).

## Ectonucleotidases

Four main groups of plasma membrane enzymes are endowed of the ability of hydrolyzing extracellular nucleotides, transforming ATP and ADP to ADO thus shifting purinergic receptor activation from P2 to P1 subtypes. Activity of ectonucleotidases is fundamental to avoid excessive accumulation of nucleotides in the extracellular *milieu* and to terminate P2 signaling (45). The following families have been described: ectonucleoside triphosphate diphosphohydrolases (NTPDases), ecto-5′-nucleotidase (CD73), ectonucleotide pyrophosphatase/phosphodiesterases (NPP) and alkaline phosphatases (12). NTPDases (among which NTPDase1 or CD39) catalyze the conversion of ATP or ADP to AMP and are highly expressed by immune cells and the vasculature (46–48). Extracellular AMP is further hydrolyzed to the anti-inflammatory ADO by CD73 (49, 50). However, the CD73 driven ADO generation has been associated with the potent suppression of anti-cancer immune responses. Thus, inhibitors of CD73 for the use in clinical practice are highly desired (51).

The proposed important immunoregulatory activities of ectonucleotidases are to prevent the development of autoimmune conditions. Accordingly, we recently observed that CD39 overexpression ameliorates experimental colitis and prevents hypoxia-related damage *in vivo* in a dextran-sulfate-sodium-induced colitis model. In addition, exogenous administration of a recombinant form of human CD39L3 (APT102) boosted the regulatory effects of endogenous CD39 *in vivo* and enhanced *in vitro* Treg functions in Crohn's disease (48). Likewise, the administration of apyrase, which has ectoenzymatic activity comparable to CD39, attenuated peribronchial eosinophilic inflammation and reduced the levels of Th2 cytokines in the bronchoalveolar lavage fluid of mice with allergic airway inflammation (52).

## Eosinophil Granulocytes

Eosinophils are granulocytes deriving from CD34^+^ bone marrow precursors expressing CD38 and CD125. Thereby, IL-3, IL-5, and GM-CSF exposure have been reported to induce eosinophil differentiation (53, 54). The differentiation process occurs in about 8 days and is mainly driven by the transcription factors GATA-1, GATA-2, c/EBP, and XBP1 (55–58). Cytokines (particularly IL-5) and chemokines (CCL11, CCL24, CCL26) promote the release of eosinophils from the bone marrow (59). After circulating in the peripheral blood for 8–12 h, mature eosinophils home into tissues (mammary gland, adipose tissue, uterus, gut, lung) where they contribute to maintaining organ integrity and promote B and T cell immune function (60–64).

Under pathophysiological conditions such as atopic diseases, rhinitis, eczema, asthma and parasitic infections, chemokine-mediated CCR3 receptor activation on eosinophils as well as the stimulation with cytokines such as IL-4, IL-5, IL-9, IL-13, GM-CSF, RANTES, MCP-3, and MCP-4 have been linked to the recruitment and accumulation of eosinophils in tissues including nasal mucosa, lungs, heart, skin, liver and bile ducts, gut and nerves (65–69). In addition, eotaxin, IL-4, and IL-13 have been shown to induce the up-regulation of the adhesion molecules VCAM-1 and PSGL-1 on epithelial cells and fibroblasts thus further promoting eosinophil trafficking and recruitment (70, 71). In contrast, IL-6, and IL-11 decrease tissue infiltration by eosinophils through inhibiting VCAM-1 expression and decreasing production of type 2 cytokines. Pro-inflammatory cytokines such as IL-1, IL-12, and TNF-α up-regulate endothelium adhesion molecules, including VCAM-1, thereby favoring eosinophil diapedesis (67, 72, 73).

**Figure 1:**
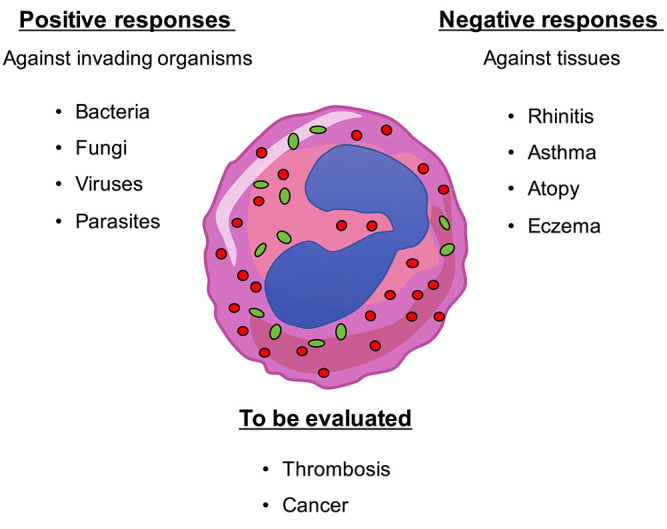
Eosinophils play multiple roles within the organism. Eosinophils are actively involved in the defense against multicellular parasites (e.g., worms) as well as fungi, bacteria, and viruses. However, they also show detrimental responses by damaging tissues and organs such as in rhinitis, asthma, atopy, eczema, etc. Involvement of eosinophils in lung tumor progression and in thrombosis have been also shown but their role has to be adequately evaluated.

Eosinophils themselves produce different cytokines including IL-1, IL-3, IL-4, IL-5, IL-6, IL-8, TNF-α, TGF-β, GM-CSF and pro-inflammatory mediators such as leukotriene C4 (LTC4), platelet-activation factor (PAF) (71, 74), the granular cationic proteins, major basic protein (MBP) 1, MBP 2, eosinophil cationic protein (ECP), eosinophil-derived neurotoxin (EDN) and eosinophil peroxidase (EPO). MBP which is present in the crystal core of the specific granules has cytotoxic effects due to interference with electrical properties and permeability of the cell membrane. MBP also triggers degranulation of mast cells and basophils (75). ECP favors the entry of cytotoxic molecules by forming voltage-insensitive, non-selective pores in the membrane of target cells (54, 68). ECP and EDN, that belong to the ribonuclease A superfamily, kill single-stranded RNA pneumoviruses (76). In addition, eosinophils generate reactive oxygen species, hypohalous acids and lysosomal hydrolases that are toxic for bacteria and parasites but also for surrounding tissues (77–80) (Figure 1).

## P2 Receptors Expressed by Eosinophils

There is currently no systematic study on the expression of P2 receptor specific mRNAs and proteins in eosinophils. Another source of uncertainty is represented by the fact that the expression of individual P2 subtypes is not replicated in all studies. This bias can be due to the presence (or absence) of contaminating cells in different eosinophil preparations and/or to sensitivity of the techniques used. Different studies revealed that human, murine and rat eosinophils express mRNAs for different P2X and P2Y receptors including P2X1, P2X4, P2X5, P2X7, P2Y1, P2Y2, P2Y4, P2Y6, P2Y11 (81–85). An RNASeq study confirmed expression of P2Y6 and P2X5 mRNAs (86). Proteomic studies have shed light on expression of P2 receptor proteins, showing that human blood eosinophils express P2Y2, P2Y4, P2Y13 and P2Y14 as well as P2X1, P2X2 (87). Several pharmacologic studies performed with P2 receptor agonists and antagonists confirmed functionality of the receptors on eosinophils. Interestingly, currents evoked by the P2X agonist alpha, beta-methylene ATP were lower in eosinophils derived from asthmatic subjects compared to eosinophils derived from healthy donors, although P2X1 mRNA and protein expression was comparable in both groups. However, this effect in eosinophils isolated from asthmatics was negated by pharmacological degradation of extracellular ATP using apyrase, suggesting that P2X1 receptors were partially desensitized due to ATP release by eosinophils and raising the question why eosinophils from asthmatic subjects might release the nucleotide (88).

## Nucleotide Mediated Responses in Eosinophils

Eosinophils are activated by a plethora of soluble mediators including cytokines such as IL-3, IL-5, IL-8, and GM-CSF, CC- chemokines, complement factors C3a and C5a, PAF, prostaglandin D2 (PGD2) and LPA, leukotriene B4 (LTB4) (59, 66, 89–91). Moreover, eosinophils respond to alarmins released by damaged tissue during infection or inflammation and stimulate immune responses and tissue remodeling (92). Nucleotide stimulation of human eosinophils was reported almost 30 years ago when it was shown that extracellular ATP secreted by thrombin-stimulated platelets exerted chemoattractant effects on human eosinophils (93, 94). Of note, the interaction of platelets and eosinophils contributing to tissue inflammation and remodeling was demonstrated in later studies (95–97).

**Figure 2:**
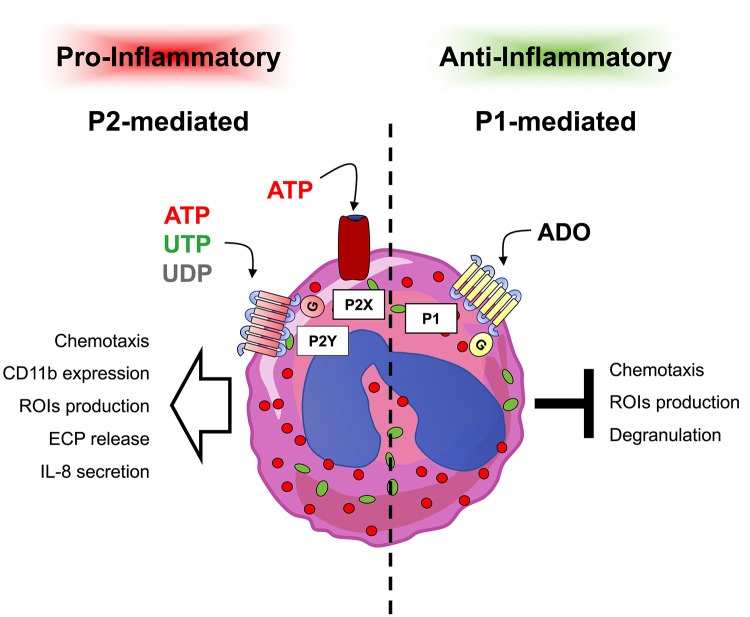
Responses induced by extracellular nucleotides and adenosine in eosinophils. Eosinophils express P1 and P2 receptor subtypes whose stimulation has been linked to different responses. In particular, pro-inflammatory P2-mediated responses (left part) confer to eosinophils a pro-inflammatory behavior, while on the contrary, anti-inflammatory P1-mediated responses (right part) induce anti-inflammatory effects.

In addition, eosinophils are also capable of secreting ATP which in turn autocrinally stimulates the release of different pro-inflammatory mediators by activating P2Y2 receptors (98) (Figure 2).

## Eosinophilic Tissue Infiltration

Airway infiltration by eosinophils is driven by binding of the cell surface molecule α4β1 integrin (VLA-4) on eosinophils to VCAM-1. Accordingly, mice deficient for VCAM-1 fail to develop pulmonary eosinophilia (99, 100). Extracellular nucleotides (ATP, UTP) have been implicated in modulating the expression and function of adhesion molecules including VCAM-1 (101, 102). In this context, P2Y2 receptor signaling might play an important role since it has been shown to modulate both membrane-bound and soluble VCAM-1 in a mouse model of OVA-induced lung inflammation. Furthermore, P2Y2-deficiency in the same model was associated with a reduced VCAM-1 up-regulation and lung eosinophilia compared to wild type animals (84).

In addition to VCAM-1, eosinophils express the integrin family member CD11b. CD11b interacts with CD18 to form the complement receptor 3 (CR3) heterodimer, which also contributes to eosinophil migration into inflamed tissue. Endothelial cells release ATP in response to different stimuli which might modulate the expression and function of CD11b and other adhesion molecules in circulating granulocytes (103, 104). Hence, *in vitro* stimulation of human eosinophils with ATP results in a fast (within seconds) and dose-dependent up-regulation of CD11b (105). In line with this, exposure of human eosinophils to pharmacological P2X and P2Y agonists induces CD11b expression (106). The fast kinetic suggests a nucleotide-mediated plasma membrane trafficking by intracellularly stored CD11b rather than an induced transcription of the *cd11b* gene which would be delayed. The P2X1 receptor subtype seems to be crucial in this context, since the P2X1 activation using alpha,beta-methylene ATP promotes αMβ2 integrin–dependent eosinophil adhesion. This effect was higher in eosinophils from healthy individuals compared to patients suffering from asthma (88).

Apart from modulating the expression of adhesion molecules, a direct chemotactic P2Y2-dependent effect of ATP on eosinophils has been demonstrated. Of note, eosinophils derived from asthmatic patients showed an up-regulation of P2Y2 receptor expression accompanied by an increased ATP-driven migration (22, 106) (Figure 2).

## Release of Effector Molecules/Pro-Inflammatory Mediators

Activated by different stimuli such as eotaxin and complement proteins C3a and C5a, human eosinophils generate reactive oxygen intermediates (ROIs). Extracellular nucleotides (ATP, ADP, UTP, GTP, and BzATP) have also been implicated in the production of ROIs via activating both P2X and P2Y receptors (83, 106). In accordance, blood eosinophils isolated from asthmatics showed an increased expression of P2X7 receptors compared to healthy controls. Simultaneously, these eosinophils produced higher amounts of ROIs after stimulation with the P2X7 agonist BzATP (107).

Eosinophil granules contain different enzymatic and non-enzymatic proteins promoting host defense but also the pathogenesis of chronic diseases such as asthma, atopic dermatitis, prurigo nodularis and vasculitis, where they cause tissue damage associated with inappropriate release. Eosinophil cationic protein (ECP) is a known marker of eosinophil activation/participation under pathophysiological conditions (108). Stimulation of eosinophils with ATP, UTP and UDP, but not BzATP, ADP or alpha,beta-methylene ATP induces the release of ECP in a dose-dependent and pertussis toxin sensitive manner. This suggests the involvement of P2Y receptors, potentially of the P2Y2 subtype, in purine-driven ECP release. Similar observations have been made for the eosinophil derived neurotoxin, a protein closely related to ECP with cytotoxic properties, which is released following P2Y2 receptor activation (83, 109). Human and mouse eosinophils also express the P2Y12 receptor. Accordingly, ADP stimulated secretion of eosinophil peroxidase (EPO) in a P2Y12 dependent manner in human eosinophils has been shown (85).

Interleukin-8 (IL-8) or CXCL8 is a human chemokine produced by innate immune cells including eosinophils but also by endothelial or epithelial cells. Augmented IL-8 secretion has been observed in eosinophils from patients with asthma or atopic dermatitis (110, 111). Human eosinophils secrete IL-8 in response to stimulation with UDP, ATP, alpha,beta-meATP, and BzATP, while UTP or ADP show no effect. From the pharmacological profile of the response and use of P2 inhibitors, both P2Y and P2X receptor subfamilies could be involved in IL-8 secretion. A participation of P2Y6, P2X1 and P2X7 subtypes has been hypothesized (109). Moreover, a recent study demonstrated that release of pro-inflammatory cytokines by human eosinophils upon stimulation with the endogenous danger signal crystalline uric acid is dependent on autocrine secretion of ATP in the extracellular space and on the expression of purinergic receptors (Figure 2) (98).

## P1 Receptors Expressed by Eosinophils and Their Responses

Adenosine P1 receptors have been shown to strongly suppress eosinophil pro-inflammatory functions. In asthma, the anti-inflammatory effects of the drug theophylline are enhanced by A3 receptors expressed on eosinophils. Accordingly, ADO administration boosts the beneficial effects of clinically relevant theophylline concentrations, while administration of the selective A3 antagonist MRS 1220 alleviates the anti-inflammatory effects of theophylline. However, A1 and A2 antagonists fail to inhibit theophylline treatment (112).

Furthermore, A3 activation in eosinophils triggers Ca^2+^ release from intracellular stores (113, 114). However, A3 activation does not appear to be a prime mechanism for free radical generation by human peripheral blood eosinophils and an inhibitory effect of A3 receptor subtypes on the degranulation of human eosinophils and O^2−^ release has been suggested (115, 116). The same adenosine receptor has been found to have a regulatory function on the migration of eosinophils to the site of inflammation. *In vitro* experiments revealed that the A3 receptor signaling inhibits the migration of human eosinophils in response to PAF, RANTES, and LTB4 (117). This inhibitory effect has been confirmed *in vivo*, where A3 activation significantly reduces PAF-induced eosinophil migration to the lungs. This suggests the use of A3 receptor agonists as a therapeutic approach for asthma and rhinitis (118). Of note, an atypical form of the A3 receptor found in human eosinophils is positively coupled to AC and promotes anti-inflammatory responses by inducing cAMP (113). In some of these human studies, expression of A3 receptor in eosinophils was determined at the mRNA level (113, 118) or by immune-labeling (116); while in most of these earlier investigations presence of A3 receptor was indirectly proven by functional or inhibition studies using selective agents or antagonists (112, 115, 117), without the expression of the receptor being proven *per se*. In recent studies, expression of ADORA2B in human eosinophils was detected by RNA-seq analysis that, however, did not detect presence of A3 receptor in the same samples (86). Further, a subsequent proteomic study by Wilkerson and colleagues, did not detect presence of P1 receptors in human eosinophils obtained from the peripheral blood. These discrepancies might result from differences in the eosinophil purification protocols or, alternatively, in the specificity of the techniques used. Further studies are needed to combine eosinophil RNA and proteomic profiling along with functional investigations, to resolve these apparent incongruities.

Under certain circumstances, selected adenosine receptors are also responsive to inosine, a purine formed by deamination and breakdown of adenosine. It has been shown that inosine contributes to lung recruitment of eosinophils in a murine model of allergic OVA-induced respiratory inflammation in an A2A- and A3-dependent manner (119) (Figure 2).

## Nucleotide Metabolizing Enzymes Expressed by Eosinophils

Information on expression and function of ectonucleotidases in human eosinophils are lacking.

Although expression of CD39 (ectonucleotidase-1) was demonstrated in human leukocytes from sputum and BALF, and its activity was shown to be modulated by smoking and increased in chronic obstructive pulmonary disease (COPD) (120), no clear attribution of CD39 protein to human eosinophil cells has been done so far.

In the context of asthma, some studies suggest a protective effect of global (or on regulatory T cells) CD39 expression in the modulation of eosinophil functions.

In the settings of ovalbumin-induced allergic airway inflammation, systemic CD39 inhibition by ARL67156 or through genetic deletion in regulatory T cells, worsens animal clinical conditions. Interestingly, control mice (i.e., mice with normal CD39 expression levels), present milder airway inflammation, associated with significantly lower eosinophil counts in BALF (121).

A correlation between CD39 levels in the thymus and eosinophil infiltration in the BALF has been recently observed also in experimental house dust mite (HDM)-induced allergic asthma. Results from this study suggest the beneficial effects of multiple doses of adipose tissue-derived mesenchymal stromal cells (MSCs). Notably, animals exposed to three doses of MSCs present significantly reduced inflammation in the lungs, this being associated to increased levels of CD39 in the thymus and lower eosinophil counts in the BALF (122).

## Conclusions

While in healthy subjects the number of eosinophils in the peripheral blood is low, it can increase dramatically under pathophysiological conditions such as atopic dermatitis, bronchial asthma, eosinophilic esophagitis, gastritis, gastroenteritis, colitis or hematological malignancies (54, 62, 71, 80, 123). The large body of evidence supporting the critical role of eosinophils in parasitic and inflammatory diseases has prompted and intensified investigations on potential targets to modulate cellular responses of eosinophils.

Inflammation is associated with the release of nucleotides in the extracellular space, where they serve as ligands for purinergic receptors. Purinergic signaling represents an ubiquitous signal transduction and regulatory system (7). Eosinophils express a wide range of purinergic receptors and purinergic receptor activation is associated with the recruitment of eosinophils into inflamed tissue, ROIs production, the release of effector molecules and the secretion of pro-inflammatory cytokines. Thus, the inhibition of purinergic receptors on eosinophils would be highly desirable for reducing detrimental immune responses and tissue damage related to various disorders. In accordance, blocking P2 receptors signaling using specific inhibitors or P2 receptor deficiency could be associated with decreased eosinophilic inflammation in diverse animal models. Given the wide range of specific P2 receptor inhibitors available and the successful application in clinical trials, further research in P2 inhibition as therapeutic strategy for treating eosinophilic diseases in humans is warranted.

Besides targeting eosinophil migration and activation, in inflammation another potential approach is the modulation of eosinophil-platelets interactions. In asthma patients it has been demonstrated that platelets bind to eosinophils in the blood. This event directly correlates with the occurrence of spontaneous or clinically induced (e.g., allergen challenge) asthmatic attacks (124). One randomized, placebo-controlled clinical study on the use of the anti-P2Y12 platelet inhibitor “Prasugrel” in asthmatic patients, has shown a slight, although not significant reduction, in the bronchial inflammatory burden (125).

However, other P2Y12 inhibitors (used for the treatment of thrombosis) have failed to control eosinophil recruitment in animal models of allergic inflammation (126), suggesting different levels in platelet activity in response to vascular injury, when compared to allergic responses (127). Furthermore, different studies confirmed the involvement of P2Y1 and P2Y14 receptors in platelet-dependent eosinophil recruitment in the lungs (126, 128, 129).

New developments of effective treatments for eosinophilic diseases, like asthma or allergy, are also important because eosinophils are a major source of intravascular tissue factor, a key initiator of blood coagulation (130). Disorders characterized by eosinophil accumulation have been associated with an increased risk of thrombosis. A study conducted on a cohort of patients affected by hypereosinophilia confirmed the presence of increased tissue factor expression in eosinophils from these patients compared to healthy controls (131). However, further investigation is needed to confirm whether this finding is truly associated with an increased risk of thrombosis. A comprehensive profiling of eosinophil P1 and P2 receptor expression pattern at both mRNA and protein levels would shed light on the function of these receptors in eosinophils, as well as on their biology and contribution to the regulation of pathologically relevant eosinophil responses.

Moreover, differences in the purinergic signaling of different eosinophil subpopulations could exist and be important for diseases where eosinophil participation is predominant (132, 133). Therefore, isolation of eosinophils subpopulations and analysis of their purinergic network would be requested. Another prerequisite is the characterization of the complete panel of cytokine/chemokines released by eosinophils in response to nucleotide stimulation. In future studies, it would be relevant to check the effect of ATP and other nucleotides on production of eosinophil preeminent cytokines and chemokines such as IL-5, eotaxin and RANTES. Further efforts should be done to elucidate expression and function of ectonucleotidases CD39 and CD73 in human eosinophils; this would give a more complete picture of the purinergic signaling of these cells and would help to interpret relationships between purinergic signaling in eosinophils and other cell types involved in the immune response and tissue remodeling. Eosinophils are thought to play either positive or negative roles in cancer, depending on type of tumor (59). Since nucleosides and nucleotides are present in the tumor microenvironment and heavily affect immune response against cancer, it would be worthy to check whether stimulation of the purinergic network of eosinophils modulate their responses against tumors.

## Author Contributions

DF, MV, ML, SR, AZ, TM, and MI conceived the review and wrote the manuscript. FC prepared the figures, contribute and revise the manuscript. EM and PS checked and revised the manuscript. All authors contributed to the article and approved the submitted version.

## Conflict of Interest

The authors declare that the research was conducted in the absence of any commercial or financial relationships that could be construed as a potential conflict of interest.

## Abbreviations

AC: adenylate cyclase
ADO: adenosine
ADP: adenosine diphosphate
ATP: adenosine triphosphate
BALF: bronchoalveolar lavage fluid
BzATP: 2',3'-O-(4-benzoyl-benzoyl)ATP
C3a: complement factor 3a
C5a: complement factor 5a
CCL11: (eotaxin-1)
CCL24: (eotaxin-2)
CCL26: (eotaxin-3)
CNS: central nervous system
COPD: chronic obstructive pulmonary disease
CR: complement receptor
DAMP: damage-associated molecular pattern
ECP: eosinophil cationic protein
EDN: eosinophil-derived neurotoxin
EPO: eosinophil peroxidase
EPO: eosinophil peroxidase
GM-CSF: granulocyte macrophage-colony stimulating factor
GTP: guanosine triphosphate
HDM: house dust mite
ICAM-1: intercellular adhesion molecule-1
IL: interleukin
LPS: lipopolysaccharides
LT: leukotriene
MBP: major basic protein
MCP: monocyte chemoattractant protein
OVA: ovalbumin
PAF: platelet-activating factor
PGD2: prostaglandin D2
MSCs: mesenchymal stromal cells
PSGL-1: P-selectin Glycoprotein Ligand-1
ROIs: reactive oxygen intermediates
TGF-β: transforming growth factor β
TNF-α: tumor necrosis factor α
UDP: uridine diphosphate
UTP: uridine triphosphate
VCAM-1: vascular cell adhesion molecule 1.
